# Supplementary material for: The interaction between sleep patterns and oxidative balance scores on the risk of cognitive function decline: Results from the national health and nutrition examination survey 2011–2014
Source: PLoS One. 2024 Dec 27;19(12):e0313784. doi: 10.1371/journal.pone.0313784 (PMC11676575; doi:10.1371/journal.pone.0313784)
Supplement: S1 Table — (DOCX) [file pone.0313784.s001.docx]

| **Table S1 Components of the OBS.** | | | | | | | |
| --- | --- | --- | --- | --- | --- | --- | --- |
| **OBS components** | **Property** | **Male** | | | **Female** | | |
|  |  | **0** | **1** | **2** | **0** | **1** | **2** |
| Dietary OBS components | | | | | | | |
| Dietary fiber (g/d) | A | <13.15 | 13.15-20.65 | ≥20.65 | <11.73 | 11.73-17.50 | ≥17.50 |
| Carotene (RE/d) | A | <266.00 | 266.00-485.95 | ≥485.95 | <229.77 | 229.77-416.50 | ≥416.50 |
| Riboflavin (mg/d) | A | <1.62 | 1.62-2.35 | ≥2.35 | <1.35 | 1.35-1.91 | ≥1.91 |
| Niacin (mg/d) | A | <19.62 | 19.62-27.88 | ≥27.88 | <15.18 | 15.18-21.39 | ≥21.39 |
| Vitamin B_6_ (mg/d) | A | <1.59 | 1.59-2.36 | ≥2.36 | <1.28 | 1.28-1.88 | ≥1.88 |
| Total folate (mcg/d) | A | <298.28 | 298.28-454.72 | ≥454.72 | <254.54 | 254.54-369.50 | ≥369.50 |
| Vitamin B_12_ (mcg/d) | A | <3.32 | 3.32-6.38 | ≥6.38 | <2.74 | 2.74-5.11 | ≥5.11 |
| Vitamin C (mg/d) | A | <43.10 | 43.10-97.66 | ≥97.66 | <42.88 | 42.88-96.19 | ≥96.19 |
| Vitamin E (ATE) (mg/d) | A | <5.97 | 5.97-9.32 | ≥9.32 | <5.18 | 5.18-8.47 | ≥8.47 |
| Calcium (mg/d) | A | <650.78 | 650.78-1011.22 | ≥1011.22 | <594.77 | 594.77-872.23 | ≥872.23 |
| Magnesium (mg/d) | A | <241.78 | 241.78-340.50 | ≥340.50 | <202.50 | 202.50-282.96 | ≥282.96 |
| Zinc (mg/d) | A | <8.55 | 8.55-12.40 | ≥12.40 | <6.68 | 6.68-9.69 | ≥9.69 |
| Copper (mg/d) | A | <0.97 | 0.97-1.37 | ≥1.37 | <0.84 | 0.84-1.19 | ≥1.19 |
| Selenium (mcg/d) | A | <91.28 | 91.28-128.37 | ≥128.37 | <70.53 | 70.53-99.75 | ≥99.75 |
| Total fat (g/d) | P | ≥88.42 | 59.34-88.42 | <59.34 | ≥70.40 | 46.01-70.40 | <46.01 |
| Iron (mg/d) | P | ≥17.11 | 11.60-17.11 | <11.60 | ≥13.60 | 9.44-13.60 | <9.44 |
| Lifestyle OBS components | | | | |  |  |  |
| Physical activity | A | Low | Moderate | High | Low | Moderate | High |
| Alcohol intake (g/d) | P | ≥30 | 0-30 | 0 | ≥15 | 0-15 | 0 |
| Body mass index (kg/m^2^) | P | Obesity | Overweight | Normal | Obesity | Overweight | Normal |
| Smoking status | P | Current  smoker | Former  smoker | Never  smoker | Current  smoker | Former  smoker | Never  smoker |
| A, antioxidant; ATE, alpha-tocopherol equivalent; OBS, oxidative balance score; P, pro-oxidant; RE, retinol equivalent. | | | | | | | |
