# Supplementary material for: The interaction between sleep patterns and oxidative balance scores on the risk of cognitive function decline: Results from the national health and nutrition examination survey 2011–2014
Source: PLoS One. 2024 Dec 27;19(12):e0313784. doi: 10.1371/journal.pone.0313784 (PMC11676575; doi:10.1371/journal.pone.0313784)
Supplement: S2 Table — (DOCX) [file pone.0313784.s002.docx]

| **Table S2. Characteristics of weighted study participants according to sleep patterns, NHANES 2011 to 2014 (Non-weighted n = 2249).** | | | | | | | | |
| --- | --- | --- | --- | --- | --- | --- | --- | --- |
|  | | **Sleep duration** | | | | **Sleep disorder** | | |
| **Characteristic** | **Overall**, N = 2249 (100%)^2^ | **Normal (7-8h)**, N = 1269 (62%)^2^ | **Short (≤6h)**, N = 748 (27%)^2^ | **Long(≥9h)**, N = 232 (11%)^2^ | ***P* Value**^3^ | **Yes**, N = 276 (12%)^2^ | **No**, N = 1973 (88%)^2^ | ***P* Value**^3^ |
| **Gender** |  |  |  |  | 0.6 |  |  | **0.010*** |
| *Male* | 1,075 (45%) | 613 (46%) | 345 (43%) | 117 (47%) |  | 153 (55%) | 922 (44%) |  |
| *Female* | 1,174 (55%) | 656 (54%) | 403 (57%) | 115 (53%) |  | 123 (45%) | 1,051 (56%) |  |
| **Age (years)** |  |  |  |  | **0.006**** |  |  | **0.034*** |
| *60-65* | 833 (40%) | 468 (41%) | 305 (41%) | 60 (30%) |  | 109 (40%) | 724 (40%) |  |
| *66-70* | 509 (22%) | 271 (21%) | 194 (26%) | 44 (20%) |  | 79 (29%) | 430 (21%) |  |
| *71-75* | 366 (16%) | 205 (15%) | 121 (17%) | 40 (18%) |  | 43 (15%) | 323 (16%) |  |
| *76-80* | 541 (22%) | 325 (22%) | 128 (16%) | 88 (32%) |  | 45 (15%) | 496 (23%) |  |
| **Race** |  |  |  |  | **<0.001***** |  |  | 0.4 |
| *Mexican American* | 186 (3%) | 103 (3%) | 67 (4%) | 16 (3%) |  | 19 (2%) | 167 (3%) |  |
| *Other Hispanic* | 204 (3%) | 107 (3%) | 87 (5%) | 10 (2%) |  | 24 (3%) | 180 (3%) |  |
| *Non-Hispanic White* | 1,153 (82%) | 718 (85%) | 283 (72%) | 152 (87%) |  | 153 (84%) | 1,000 (82%) |  |
| *Non-Hispanic Black* | 525 (8%) | 244 (6%) | 237 (13%) | 44 (7%) |  | 65 (8%) | 460 (8%) |  |
| *Other Race(Including Multi-Racial)* | 181 (4%) | 97 (3%) | 74 (7%) | 10 (2%) |  | 15 (3%) | 166 (4%) |  |
| **Education level** |  |  |  |  | **0.045*** |  |  | 0.2 |
| *Less than 9th grade* | 209 (4.9%) | 108 (4.0%) | 77 (5.6%) | 24 (8.1%) |  | 18 (3.7%) | 191 (5.0%) |  |
| *9-11th grade (Includes 12th grade with no diploma)* | 299 (9.8%) | 163 (9.4%) | 106 (11%) | 30 (7.9%) |  | 37 (11%) | 262 (9.7%) |  |
| *High school graduate/GED or equivalent* | 526 (21%) | 291 (21%) | 183 (23%) | 52 (20%) |  | 58 (16%) | 468 (22%) |  |
| *Some college/AA degree* | 660 (32%) | 375 (32%) | 222 (35%) | 63 (28%) |  | 97 (41%) | 563 (31%) |  |
| *College graduate or above* | 553 (32%) | 331 (34%) | 159 (25%) | 63 (36%) |  | 66 (29%) | 487 (32%) |  |
| *Don’t know/Refused* | 2 (<0.1%) | 1 (<0.1%) | 1 (<0.1%) | 0 (0%) |  | 0 (0%) | 2 (<0.1%) |  |
| **Marital status** |  |  |  |  | **0.009**** |  |  | 0.5 |
| *Married/Living with partner* | 1,315 (66%) | 783 (70%) | 402 (57%) | 130 (63%) |  | 170 (67%) | 1,145 (66%) |  |
| *Widowed/Divorced/Separated* | 808 (30%) | 427 (26%) | 298 (38%) | 83 (32%) |  | 95 (31%) | 713 (30%) |  |
| *Never married* | 125 (4.2%) | 59 (3.7%) | 47 (4.9%) | 19 (5.0%) |  | 11 (2.6%) | 114 (4.4%) |  |
| *Don’t know/Refused* | 1 (<0.1%) | 0 (0%) | 1 (<0.1%) | 0 (0%) |  | 0 (0%) | 1 (<0.1%) |  |
| **Ratio of family income to poverty** |  |  |  |  | **0.020*** |  |  | 0.6 |
| *PIR＜1.3* | 622 (16%) | 320 (14%) | 222 (20%) | 80 (18%) |  | 84 (18%) | 538 (16%) |  |
| *1.3≤PIR＜3.5* | 884 (39%) | 501 (39%) | 303 (41%) | 80 (38%) |  | 101 (40%) | 783 (39%) |  |
| *PIR≥3.5* | 743 (45%) | 448 (48%) | 223 (39%) | 72 (45%) |  | 91 (42%) | 652 (45%) |  |
| **Diabetes** |  |  |  |  | **0.003**** |  |  | **<0.001***** |
| *Yes* | 736 (26%) | 388 (24%) | 273 (31%) | 75 (29%) |  | 131 (43%) | 605 (24%) |  |
| *No* | 1,513 (74%) | 881 (76%) | 475 (69%) | 157 (71%) |  | 145 (57%) | 1,368 (76%) |  |
| **Hyperlipidemia** |  |  |  |  | 0.2 |  |  | **0.010*** |
| *Yes* | 1,876 (84%) | 1,063 (85%) | 620 (82%) | 193 (87%) |  | 246 (91%) | 1,630 (84%) |  |
| *No* | 373 (16%) | 206 (15%) | 128 (18%) | 39 (13%) |  | 30 (9.3%) | 343 (16%) |  |
| **Depression** |  |  |  |  | **<0.001***** |  |  | **0.004**** |
| *Yes* | 201 (7.2%) | 77 (5.0%) | 106 (13%) | 18 (5.5%) |  | 55 (14%) | 146 (6.3%) |  |
| *No* | 2,048 (93%) | 1,192 (95%) | 642 (87%) | 214 (94%) |  | 221 (86%) | 1,827 (94%) |  |
| **Hypertension** |  |  |  |  | 0.073 |  |  | 0.2 |
| *Yes* | 1,576 (65%) | 858 (63%) | 544 (68%) | 174 (74%) |  | 207 (70%) | 1,369 (65%) |  |
| *No* | 673 (35%) | 411 (37%) | 204 (32%) | 58 (26%) |  | 69 (30%) | 604 (35%) |  |
| **OBS** |  |  |  |  | 0.4 |  |  | **0.025*** |
| *Q1* | 593 (22%) | 304 (20%) | 223 (24%) | 66 (26%) |  | 91 (31%) | 502 (21%) |  |
| *Q2* | 604 (25%) | 338 (25%) | 207 (26%) | 59 (23%) |  | 72 (25%) | 532 (25%) |  |
| *Q3* | 544 (26%) | 317 (26%) | 169 (26%) | 58 (27%) |  | 60 (23%) | 484 (27%) |  |
| *Q4* | 508 (27%) | 310 (28%) | 149 (24%) | 49 (23%) |  | 53 (20%) | 455 (27%) |  |
| **Cognitive Performance** |  |  |  |  |  |  |  |  |
| **DSST** |  |  |  |  | **<0.001***** |  |  | 0.5 |
| *Normal (≥34)* | 1,755 (88%) | 1,002 (90%) | 588 (87%) | 165 (80%) |  | 226 (89%) | 1,529 (88%) |  |
| *Poor (<34)* | 494 (12%) | 267 (10%) | 160 (13%) | 67 (20%) |  | 50 (11%) | 444 (12%) |  |
| **CERAD-WL** |  |  |  |  | **0.022*** |  |  | 0.6 |
| *Normal (≥17)* | 1,653 (80%) | 946 (81%) | 557 (79%) | 150 (72%) |  | 208 (81%) | 1,445 (79%) |  |
| *Poor (<17)* | 596 (20%) | 323 (19%) | 191 (21%) | 82 (28%) |  | 68 (19%) | 528 (21%) |  |
| **CERAD-DR** |  |  |  |  | **<0.001***** |  |  | 0.3 |
| *Normal (≥5)* | 1,719 (80%) | 965 (80%) | 603 (85%) | 151 (65%) |  | 218 (83%) | 1,501 (79%) |  |
| *Poor (<5)* | 530 (20%) | 304 (20%) | 145 (15%) | 81 (35%) |  | 58 (17%) | 472 (21%) |  |
| **AF** |  |  |  |  | **<0.001***** |  |  | 0.3 |
| *Normal (≥14)* | 1,624 (81%) | 956 (83%) | 522 (79%) | 146 (71%) |  | 206 (83%) | 1,418 (80%) |  |
| *Poor (<14)* | 625 (19%) | 313 (17%) | 226 (21%) | 86 (29%) |  | 70 (17%) | 555 (20%) |  |
| ^1^N not Missing | | | | | | | | |
| ^2^median (IQR) for continuous; n (%) for categorical | | | | | | | | |
| ^3^Wilcoxon rank-sum test for complex survey samples; chi-squared test with Rao & Scott's second-order correction | | | | | | | | |
| PIR – poverty-income ratio, OBS – oxidative balance score, Q – quartile | | | | | | | | |
| *P < 0.05,**P<0.01,***P<0.001. | | | | | | | | |
