# Supplementary material for: The interaction between sleep patterns and oxidative balance scores on the risk of cognitive function decline: Results from the national health and nutrition examination survey 2011–2014
Source: PLoS One. 2024 Dec 27;19(12):e0313784. doi: 10.1371/journal.pone.0313784 (PMC11676575; doi:10.1371/journal.pone.0313784)
Supplement: S3 Table — (DOCX) [file pone.0313784.s003.docx]

| **Table S3. Characteristics of weighted study participants according to OBS, NHANES 2011 to 2014 (Non-weighted n = 2249).** | | | | | | |
| --- | --- | --- | --- | --- | --- | --- |
|  | | **OBS** | | | | |
| **Characteristic** | **Overall**, N = 2249 (100%)^2^ | **Q1**, N = 593 (22%)^2^ | **Q2**, N = 604 (25%)^2^ | **Q3**, N = 544 (26%)^2^ | **Q4**, N = 508 (27%)^2^ | ***P* Value**^3^ |
| **Gender** |  |  |  |  |  | **0.005**** |
| *Male* | 1,075 (45%) | 268 (41%) | 274 (41%) | 258 (45%) | 275 (54%) |  |
| *Female* | 1,174 (55%) | 325 (59%) | 330 (59%) | 286 (55%) | 233 (46%) |  |
| **Age (years)** |  |  |  |  |  | 0.15 |
| *60-65* | 833 (40%) | 207 (35%) | 213 (39%) | 208 (43%) | 205 (43%) |  |
| *66-70* | 509 (22%) | 170 (29%) | 121 (20%) | 114 (22%) | 104 (20%) |  |
| *71-75* | 366 (16%) | 81 (14%) | 112 (18%) | 84 (14%) | 89 (18%) |  |
| *76-80* | 541 (22%) | 135 (22%) | 158 (23%) | 138 (22%) | 110 (20%) |  |
| **Race** |  |  |  |  |  | **<0.001***** |
| *Mexican American* | 186 (3%) | 51 (4%) | 41 (3%) | 52 (3%) | 42 (2%) |  |
| *Other Hispanic* | 204 (3%) | 55 (4%) | 65 (4%) | 51 (3%) | 33 (2%) |  |
| *Non-Hispanic White* | 1,153 (82%) | 249 (74%) | 301 (81%) | 303 (84%) | 300 (87%) |  |
| *Non-Hispanic Black* | 525 (8%) | 201 (14%) | 147 (9%) | 99 (6%) | 78 (4%) |  |
| *Other Race(Including Multi-Racial)* | 181 (4%) | 37 (4%) | 50 (4%) | 39 (4%) | 55 (4%) |  |
| **Education level** |  |  |  |  |  | **<0.001***** |
| *Less than 9th grade* | 209 (4.9%) | 81 (9.4%) | 58 (5.5%) | 50 (4.3%) | 20 (1.2%) |  |
| *9-11th grade (Includes 12th grade with no diploma)* | 299 (9.8%) | 108 (14%) | 84 (11%) | 55 (7.4%) | 52 (8.0%) |  |
| *High school graduate/GED or equivalent* | 526 (21%) | 153 (24%) | 160 (24%) | 117 (21%) | 96 (17%) |  |
| *Some college/AA degree* | 660 (32%) | 157 (32%) | 178 (34%) | 170 (34%) | 155 (30%) |  |
| *College graduate or above* | 553 (32%) | 92 (21%) | 124 (26%) | 152 (33%) | 185 (44%) |  |
| *Don’t know/Refused* | 2 (<0.1%) | 2 (<0.1%) | 0 (0%) | 0 (0%) | 0 (0%) |  |
| **Marital status** |  |  |  |  |  | **0.031*** |
| *Married/Living with partner* | 1,315 (66%) | 323 (59%) | 335 (61%) | 327 (69%) | 330 (73%) |  |
| *Widowed/Divorced/Separated* | 808 (30%) | 229 (36%) | 238 (35%) | 190 (27%) | 151 (23%) |  |
| *Never married* | 125 (4.2%) | 40 (5.1%) | 31 (3.9%) | 27 (3.6%) | 27 (4.1%) |  |
| *Don’t know/Refused* | 1 (<0.1%) | 1 (<0.1%) | 0 (0%) | 0 (0%) | 0 (0%) |  |
| **Ratio of family income to poverty** |  |  |  |  |  | **<0.001***** |
| *PIR＜1.3* | 622 (16%) | 213 (25%) | 177 (18%) | 129 (12%) | 103 (11%) |  |
| *1.3≤PIR＜3.5* | 884 (39%) | 255 (48%) | 243 (40%) | 210 (38%) | 176 (33%) |  |
| *PIR≥3.5* | 743 (45%) | 125 (28%) | 184 (42%) | 205 (50%) | 229 (57%) |  |
| **Diabetes** |  |  |  |  |  | **0.004**** |
| *Yes* | 736 (26%) | 244 (34%) | 206 (29%) | 159 (25%) | 127 (18%) |  |
| *No* | 1,513 (74%) | 349 (66%) | 398 (71%) | 385 (75%) | 381 (82%) |  |
| **Hyperlipidemia** |  |  |  |  |  | **0.039*** |
| *Yes* | 1,876 (84%) | 507 (86%) | 509 (85%) | 456 (87%) | 404 (80%) |  |
| *No* | 373 (16%) | 86 (14%) | 95 (15%) | 88 (13%) | 104 (20%) |  |
| **Depression** |  |  |  |  |  | **0.005**** |
| *Yes* | 201 (7.2%) | 68 (11%) | 51 (6.0%) | 44 (6.8%) | 38 (5.5%) |  |
| *No* | 2,048 (93%) | 525 (89%) | 553 (94%) | 500 (93%) | 470 (94%) |  |
| **Hypertension** |  |  |  |  |  | **0.005**** |
| *Yes* | 1,576 (65%) | 460 (76%) | 424 (64%) | 363 (64%) | 329 (58%) |  |
| *No* | 673 (35%) | 133 (24%) | 180 (36%) | 181 (36%) | 179 (42%) |  |
| **Sleep duration** |  |  |  |  |  | 0.4 |
| *Normal (7-8h)* | 1,269 (62%) | 304 (57%) | 338 (62%) | 317 (62%) | 310 (67%) |  |
| *Short (≤6h)* | 748 (27%) | 223 (30%) | 207 (28%) | 169 (27%) | 149 (24%) |  |
| *Long (≥9h)* | 232 (11%) | 66 (13%) | 59 (9.6%) | 58 (11%) | 49 (9.3%) |  |
| **Sleep disorder** |  |  |  |  |  | **0.025*** |
| *Yes* | 276 (12%) | 91 (17%) | 72 (12%) | 60 (11%) | 53 (9.1%) |  |
| *No* | 1,973 (88%) | 502 (83%) | 532 (88%) | 484 (89%) | 455 (91%) |  |
| **Cognitive Performance** |  |  |  |  |  |  |
| **DSST** |  |  |  |  |  | **<0.001***** |
| *Normal (≥34)* | 1,755 (88%) | 400 (79%) | 466 (86%) | 443 (91%) | 446 (93%) |  |
| *Poor (<34)* | 494 (12%) | 193 (21%) | 138 (14%) | 101 (8.7%) | 62 (6.7%) |  |
| **CERAD-WL** |  |  |  |  |  | **<0.001***** |
| *Normal (≥17)* | 1,653 (80%) | 405 (73%) | 434 (76%) | 406 (81%) | 408 (87%) |  |
| *Poor (<17)* | 596 (20%) | 188 (27%) | 170 (24%) | 138 (19%) | 100 (13%) |  |
| **CERAD-DR** |  |  |  |  |  | 0.12 |
| *Normal (≥5)* | 1,719 (80%) | 439 (77%) | 455 (79%) | 413 (79%) | 412 (84%) |  |
| *Poor (<5)* | 530 (20%) | 154 (23%) | 149 (21%) | 131 (21%) | 96 (16%) |  |
| **AF** |  |  |  |  |  | **<0.001***** |
| *Normal (≥14)* | 1,624 (81%) | 376 (73%) | 417 (76%) | 424 (86%) | 407 (85%) |  |
| *Poor (<14)* | 625 (19%) | 217 (27%) | 187 (24%) | 120 (14%) | 101 (15%) |  |
| ^1^N not Missing | | | | | | |
| ^2^median (IQR) for continuous; n (%) for categorical | | | | | | |
| ^3^Wilcoxon rank-sum test for complex survey samples; chi-squared test with Rao & Scott's second-order correction | | | | | | |
| PIR – poverty-income ratio, OBS – oxidative balance score, Q – quartile | | | | | | |
| *P < 0.05,**P<0.01,***P<0.001. | | | | | | |
