# Supplementary material for: The interaction between sleep patterns and oxidative balance scores on the risk of cognitive function decline: Results from the national health and nutrition examination survey 2011–2014
Source: PLoS One. 2024 Dec 27;19(12):e0313784. doi: 10.1371/journal.pone.0313784 (PMC11676575; doi:10.1371/journal.pone.0313784)
Supplement: S4 Table — (DOCX) [file pone.0313784.s004.docx]

| **Table S4. Characteristics of weighted study participants according to Cognitive Performance (CERAD, AF), NHANES 2011 to 2014 (Non-weighted n = 2249).** | | | | | | | | | | |
| --- | --- | --- | --- | --- | --- | --- | --- | --- | --- | --- |
|  | | **Cognitive Performance（CERAD-WL score）** | | | **Cognitive Performance（CERAD-DR score）** | | | **Cognitive Performance（AF score）** | | |
| **Characteristic** | **Overall**, N = 2249 (100%)^2^ | **Normal(≥17)**, N = 1653 (80%)^2^ | **Poor(<17)**, N = 596 (20%)^2^ | ***P* Value**^3^ | **Normal(≥5)**, N = 1719 (80%)^2^ | **Poor(<5)**, N = 530 (20%)^2^ | ***P* Value**^3^ | **Normal(≥14)**, N = 1624 (81%)^2^ | **Poor(<14)**, N = 625 (19%)^2^ | ***P* Value**^3^ |
| **Gender** |  |  |  | **<0.001***** |  |  | **<0.001***** |  |  | 0.6 |
| *Male* | 1,075 (45%) | 727 (43%) | 348 (55%) |  | 757 (43%) | 318 (56%) |  | 778 (46%) | 297 (44%) |  |
| *Female* | 1,174 (55%) | 926 (57%) | 248 (45%) |  | 962 (57%) | 212 (44%) |  | 846 (54%) | 328 (56%) |  |
| **Age (years)** |  |  |  | **<0.001***** |  |  | **<0.001***** |  |  | **<0.001***** |
| *60-65* | 833 (40%) | 688 (45%) | 145 (23%) |  | 714 (45%) | 119 (19%) |  | 659 (44%) | 174 (25%) |  |
| *66-70* | 509 (22%) | 399 (24%) | 110 (16%) |  | 410 (23%) | 99 (21%) |  | 375 (24%) | 134 (17%) |  |
| *71-75* | 366 (16%) | 252 (15%) | 114 (21%) |  | 274 (15%) | 92 (19%) |  | 246 (14%) | 120 (23%) |  |
| *76-80* | 541 (22%) | 314 (17%) | 227 (41%) |  | 321 (17%) | 220 (41%) |  | 344 (18%) | 197 (35%) |  |
| **Race** |  |  |  | **0.001***** |  |  | **0.041*** |  |  | **<0.001***** |
| *Mexican American* | 186 (3%) | 126 (3%) | 60 (5%) |  | 137 (3%) | 49 (4%) |  | 140 (3%) | 46 (4%) |  |
| *Other Hispanic* | 204 (3%) | 131 (2%) | 73 (6%) |  | 150 (3%) | 54 (4%) |  | 135 (2%) | 69 (6%) |  |
| *Non-Hispanic White* | 1,153 (82%) | 872 (83%) | 281 (76%) |  | 877 (82%) | 276 (80%) |  | 927 (85%) | 226 (67%) |  |
| *Non-Hispanic Black* | 525 (8%) | 388 (7%) | 137 (10%) |  | 397 (8%) | 128 (9%) |  | 311 (6%) | 214 (16%) |  |
| *Other Race(Including Multi-Racial)* | 181 (4%) | 136 (4%) | 45 (5%) |  | 158 (5%) | 23 (3%) |  | 111 (3%) | 70 (7%) |  |
| **Education level** |  |  |  | **<0.001***** |  |  | \| **0.003**** \| \| --- \| |  |  | **<0.001***** |
| *Less than 9th grade* | 209 (4.9%) | 88 (2.9%) | 121 (13%) |  | 122 (3.9%) | 87 (8.8%) |  | 112 (3.3%) | 97 (12%) |  |
| *9-11th grade (Includes 12th grade with no diploma)* | 299 (9.8%) | 193 (8.2%) | 106 (16%) |  | 217 (9.0%) | 82 (13%) |  | 180 (8.5%) | 119 (15%) |  |
| *High school graduate/GED or equivalent* | 526 (21%) | 389 (20%) | 137 (25%) |  | 389 (20%) | 137 (25%) |  | 346 (19%) | 180 (31%) |  |
| *Some college/AA degree* | 660 (32%) | 532 (34%) | 128 (26%) |  | 543 (34%) | 117 (26%) |  | 518 (34%) | 142 (25%) |  |
| *College graduate or above* | 553 (32%) | 450 (35%) | 103 (20%) |  | 447 (33%) | 106 (27%) |  | 468 (35%) | 85 (17%) |  |
| *Don’t know/Refused* | 2 (<0.1%) | 1 (<0.1%) | 1 (<0.1%) |  | 1 (<0.1%) | 1 (<0.1%) |  | 0 (0%) | 2 (0.1%) |  |
| **Marital status** |  |  |  | **0.010*** |  |  | 0.3 |  |  | **0.031*** |
| *Married/Living with partner* | 1,315 (66%) | 983 (67%) | 332 (60%) |  | 1,016 (67%) | 299 (63%) |  | 975 (68%) | 340 (59%) |  |
| *Widowed/Divorced/Separated* | 808 (30%) | 576 (28%) | 232 (36%) |  | 603 (29%) | 205 (34%) |  | 556 (28%) | 252 (36%) |  |
| *Never married* | 125 (4.2%) | 93 (4.2%) | 32 (4.2%) |  | 99 (4.3%) | 26 (3.6%) |  | 93 (4.0%) | 32 (5.0%) |  |
| *Don’t know/Refused* | 1 (<0.1%) | 1 (<0.1%) | 0 (0%) |  | 1 (<0.1%) | 0 (0%) |  | 0 (0%) | 1 (<0.1%) |  |
| **Ratio of family income to poverty** |  |  |  | **<0.001***** |  |  | **<0.001***** |  |  | **<0.001***** |
| *PIR＜1.3* | 622 (16%) | 388 (12%) | 234 (29%) |  | 438 (14%) | 184 (23%) |  | 381 (13%) | 241 (27%) |  |
| *1.3≤PIR＜3.5* | 884 (39%) | 656 (38%) | 228 (43%) |  | 676 (39%) | 208 (41%) |  | 637 (38%) | 247 (44%) |  |
| *PIR≥3.5* | 743 (45%) | 609 (49%) | 134 (28%) |  | 605 (47%) | 138 (36%) |  | 606 (49%) | 137 (29%) |  |
| **Diabetes** |  |  |  | **0.031*** |  |  | 0.13 |  |  | **0.019*** |
| *Yes* | 736 (26%) | 508 (25%) | 228 (32%) |  | 544 (25%) | 192 (30%) |  | 501 (25%) | 235 (32%) |  |
| *No* | 1,513 (74%) | 1,145 (75%) | 368 (68%) |  | 1,175 (75%) | 338 (70%) |  | 1,123 (75%) | 390 (68%) |  |
| **Hyperlipidemia** |  |  |  | 0.8 |  |  | 0.5 |  |  | 0.7 |
| *Yes* | 1,876 (84%) | 1,385 (85%) | 491 (84%) |  | 1,439 (84%) | 437 (85%) |  | 1,364 (85%) | 512 (84%) |  |
| *No* | 373 (16%) | 268 (15%) | 105 (16%) |  | 280 (16%) | 93 (15%) |  | 260 (15%) | 113 (16%) |  |
| **Depression** |  |  |  | 0.4 |  |  | >0.9 |  |  | **0.026*** |
| *Yes* | 201 (7.2%) | 135 (6.9%) | 66 (8.3%) |  | 154 (7.2%) | 47 (7.1%) |  | 123 (6.5%) | 78 (10%) |  |
| *No* | 2,048 (93%) | 1,518 (93%) | 530 (92%) |  | 1,565 (93%) | 483 (93%) |  | 1,501 (94%) | 547 (90%) |  |
| **Hypertension** |  |  |  | **<0.001***** |  |  | **0.002**** |  |  | **<0.001***** |
| *Yes* | 1,576 (65%) | 1,128 (62%) | 448 (76%) |  | 1,180 (63%) | 396 (75%) |  | 1,095 (63%) | 481 (75%) |  |
| *No* | 673 (35%) | 525 (38%) | 148 (24%) |  | 539 (37%) | 134 (25%) |  | 529 (37%) | 144 (25%) |  |
| **Sleep duration** |  |  |  | **0.022*** |  |  | **<0.001***** |  |  | **<0.001***** |
| *Normal(7-8h)* | 1,269 (62%) | 946 (63%) | 323 (58%) |  | 965 (62%) | 304 (61%) |  | 956 (64%) | 313 (54%) |  |
| *Short Sleep(≤6h)* | 748 (27%) | 557 (27%) | 191 (28%) |  | 603 (29%) | 145 (21%) |  | 522 (27%) | 226 (30%) |  |
| *Long Sleep(≥9h)* | 232 (11%) | 150 (9.6%) | 82 (14%) |  | 151 (8.6%) | 81 (18%) |  | 146 (9.2%) | 86 (16%) |  |
| **Sleep disorder** |  |  |  | 0.6 |  |  | 0.3 |  |  | 0.3 |
| *Yes* | 276 (12%) | 208 (12%) | 68 (11%) |  | 218 (12%) | 58 (10%) |  | 206 (12%) | 70 (10%) |  |
| *No* | 1,973 (88%) | 1,445 (88%) | 528 (89%) |  | 1,501 (88%) | 472 (90%) |  | 1,418 (88%) | 555 (90%) |  |
| **OBS** |  |  |  | **<0.001***** |  |  | 0.12 |  |  | **<0.001***** |
| *Q1* | 593 (22%) | 405 (20%) | 188 (28%) |  | 439 (21%) | 154 (25%) |  | 376 (20%) | 217 (30%) |  |
| *Q2* | 604 (25%) | 434 (24%) | 170 (30%) |  | 455 (25%) | 149 (27%) |  | 417 (24%) | 187 (31%) |  |
| *Q3* | 544 (26%) | 406 (27%) | 138 (25%) |  | 413 (26%) | 131 (28%) |  | 424 (28%) | 120 (19%) |  |
| *Q4* | 508 (27%) | 408 (29%) | 100 (17%) |  | 412 (28%) | 96 (21%) |  | 407 (28%) | 101 (20%) |  |
| ^1^N not Missing | | | | |  |  |  |  |  |  |
| ^2^median (IQR) for continuous; n (%) for categorical | | | | | | | | | | |
| ^3^Wilcoxon rank-sum test for complex survey samples; chi-squared test with Rao & Scott's second-order correction | | | | | | | | | | |
| PIR – poverty-income ratio, OBS – oxidative balance score, Q – quartile | | | | | | | | | | |
| *P < 0.05,**P<0.01,***P<0.001. | | | | | | | | | | |
