# Supplementary material for: The interaction between sleep patterns and oxidative balance scores on the risk of cognitive function decline: Results from the national health and nutrition examination survey 2011–2014
Source: PLoS One. 2024 Dec 27;19(12):e0313784. doi: 10.1371/journal.pone.0313784 (PMC11676575; doi:10.1371/journal.pone.0313784)
Supplement: S5 Table — (DOCX) [file pone.0313784.s005.docx]

| **Table S5. Weighted odds ratios with 95% CI for the associations between sleep patterns and PCP.** | | | | | | | | | | |
| --- | --- | --- | --- | --- | --- | --- | --- | --- | --- | --- |
| **Characteristic** | | **Crude model** | | | **Model 1** | | **Model 2** | | **Model 3** | |
|  |  | **OR**^1^(**95% CI**^1^) | | ***P*-value** | **OR**^1^(**95% CI**^1^) | ***P*-value** | **OR**^1^(**95% CI**^1^) | ***P*-value** | **OR**^1^(**95% CI**^1^) | ***P*-value** |
| **DSST<34** | **Sleep duration** |  |  | **0.002**** |  | **0.006**** |  | **0.023*** |  | **0.021*** |
|  | *Normal(7-8h)* | — | |  | — |  | — |  | — |  |
|  | *Short Sleep(≤6h)* | 1.29(0.99, 1.68) | |  | 1.11(0.76, 1.62) |  | 0.99(0.64, 1.51) |  | 0.89(0.56, 1.42) |  |
|  | *Long Sleep(≥9h)* | 2.18(1.38, 3.44) | |  | 2.02(1.27, 3.22) |  | 1.95(1.12, 3.41) |  | 1.91(1.05, 3.48) |  |
|  | **Sleep disorder** |  |  | 0.5 |  | 0.7 |  | 0.6 |  | 0.8 |
|  | *Yes* | — | |  | — |  | — |  | — |  |
|  | *No* | 1.14(0.77, 1.67) | |  | 0.92(0.61, 1.37) |  | 0.89(0.54, 1.46) |  | 1.08(0.62, 1.89) |  |
| **CERAD-WL<17** | **Sleep duration** |  | | **0.035*** |  | 0.2 |  | 0.4 |  | 0.4 |
|  | *Normal(7-8h)* | — | |  | — |  | — |  | — |  |
|  | *Short Sleep(≤6h)* | 1.13(0.88, 1.44) | |  | 1.17(0.92, 1.48) |  | 1.07(0.81, 1.41) |  | 1.06(0.79, 1.42) |  |
|  | *Long Sleep(≥9h)* | 1.64(1.11, 2.43) | |  | 1.43 (0.93, 2.21) |  | 1.35(0.85, 2.16) |  | 1.33(0.79, 2.23) |  |
|  | **Sleep disorder** |  | | 0.5 |  | >0.9 |  | 0.8 |  | 0.8 |
|  | *Yes* | — | |  | — |  | — |  | — |  |
|  | *No* | 1.12(0.76, 1.65) | |  | 1.02(0.66, 1.57) |  | 1.05(0.64, 1.72) |  | 1.07(0.61, 1.86) |  |
| **CERAD-DR<5** | **Sleep duration** |  | | **<0.001***** |  | **<0.001***** |  | **<0.001***** |  | **<0.001***** |
|  | *Normal(7-8h)* | — | |  | — |  | — |  | — |  |
|  | *Short Sleep(≤6h)* | 0.73(0.53, 1.02) | |  | 0.75(0.52, 1.08) |  | 0.72(0.48, 1.07) |  | 0.71(0.46, 1.08) |  |
|  | *Long Sleep(≥9h)* | 2.17(1.49, 3.16) | |  | 1.93(1.22, 3.05) |  | 1.88(1.16, 3.04) |  | 1.83(1.11, 3.04) |  |
|  | **Sleep disorder** |  | | 0.3 |  | 0.4 |  | 0.4 |  | 0.4 |
|  | *Yes* | — | |  | — |  | — |  | — |  |
|  | *No* | 1.23(0.80, 1.89) | |  | 1.20(0.75, 1.92) |  | 1.19(0.74, 1.93) |  | 1.22(0.73, 2.03) |  |
| **AF<14** | **Sleep duration** |  | | **0.002**** |  | **0.004**** |  | **0.017*** |  | **0.014*** |
|  | *Normal(7-8h)* | — | |  | — |  | — |  | — |  |
|  | *Short Sleep(≤6h)* | 1.37(1.04, 1.81) | |  | 1.22(0.86, 1.71) |  | 1.14(0.79, 1.64) |  | 1.11(0.76, 1.63) |  |
|  | *Long Sleep(≥9h)* | 2.08(1.36, 3.17) | |  | 1.93(1.28, 2.92) |  | 1.90(1.16, 3.12) |  | 1.89(1.13, 3.16) |  |
|  | **Sleep disorder** |  | | 0.3 |  | 0.4 |  | 0.4 |  | 0.4 |
|  | *Yes* | — | |  | — |  | — |  | — |  |
|  | *No* | 1.23(0.80, 1.89) | |  | 1.20(0.75, 1.92) |  | 1.19(0.74, 1.93) |  | 1.22(0.73, 2.03) |  |
| ^1^OR = Odds Ratio, CI = Confidence Interval | | | | | | | | | | |
| *P < 0.05,**P<0.01,***P<0.001. | | | | | | | | | | |
