# Supplementary material for: The interaction between sleep patterns and oxidative balance scores on the risk of cognitive function decline: Results from the national health and nutrition examination survey 2011–2014
Source: PLoS One. 2024 Dec 27;19(12):e0313784. doi: 10.1371/journal.pone.0313784 (PMC11676575; doi:10.1371/journal.pone.0313784)
Supplement: S6 Table — (DOCX) [file pone.0313784.s006.docx]

| **Table S6. Weighted odds ratios with 95% CI for the associations between OBS and PCP.** | | | | | | | | | |
| --- | --- | --- | --- | --- | --- | --- | --- | --- | --- |
| **Characteristic** | | **Crude model** | | **Model 1** | | **Model 2** | | **Model 3** | |
|  |  | **OR**^1^(**95% CI**^1^) | ***P*-value** | **OR**^1^(**95% CI**^1^) | ***P*-value** | **OR**^1^(**95% CI**^1^) | ***P*-value** | **OR**^1^(**95% CI**^1^) | ***P*-value** |
| **DSST<34** | **OBS(quartile)** |  | **<0.001***** |  | **<0.001***** |  | **0.001**** |  | **0.004**** |
|  | *Q1* | — |  | — |  | — |  | — |  |
|  | *Q2* | 0.58(0.42, 0.80) |  | 0.58(0.39, 0.87) |  | 0.66(0.41, 1.07) |  | 0.70(0.42, 1.18) |  |
|  | *Q3* | 0.35(0.22, 0.55) |  | 0.36(0.22, 0.60) |  | 0.46(0.27, 0.80) |  | 0.50(0.27, 0.91) |  |
|  | *Q4* | 0.26(0.16, 0.43) |  | 0.30(0.19, 0.48) |  | 0.48(0.27, 0.83) |  | 0.50(0.29, 0.92) |  |
|  | **OBS(median)** |  | **<0.001***** |  | **<0.001***** |  | **<0.001***** |  | **<0.001***** |
|  | *Pro-oxidative* | — |  | — |  | — |  | — |  |
|  | *Anti-oxidative* | 0.40(0.30, 0.54) |  | 0.44(0.34, 0.57) |  | 0.58(0.43, 0.79) |  | 0.60(0.44, 0.83) |  |
| **CERAD-WL<17** | **OBS(quartile)** |  | **<0.001***** |  | **<0.001***** |  | **<0.001***** |  | **<0.001***** |
|  | *Q1* | — |  | — |  | — |  | — |  |
|  | *Q2* | 0.89(0.63, 1.25) |  | 0.85(0.56, 1.29) |  | 0.96(0.63, 1.47) |  | 1.00(0.62, 1.60) |  |
|  | *Q3* | 0.65(0.41, 1.04) |  | 0.64(0.37, 1.12) |  | 0.80(0.47, 1.37) |  | 0.82(0.46, 1.48) |  |
|  | *Q4* | 0.41(0.29, 0.59) |  | 0.38(0.25, 0.58) |  | 0.50(0.33, 0.74) |  | 0.50(0.29, 0.92) |  |
|  | **OBS(median)** |  | **<0.001***** |  | **<0.001***** |  | **<0.002**** |  | **<0.002**** |
|  | *Pro-oxidative* | — |  | — |  | — |  | — |  |
|  | *Anti-oxidative* | 0.56(0.43, 0.72) |  | 0.55(0.40, 0.74) |  | 0.66(0.50, 0.88) |  | 0.66(0.49, 0.90) |  |
| **CERAD-DR<5** | **OBS(quartile)** |  | 0.14 |  | 0.2 |  | 0.4 |  | 0.4 |
|  | *Q1* | — |  | — |  | — |  | — |  |
|  | *Q2* | 0.92(0.65, 1.30) |  | 0.93(0.61, 1.40) |  | 0.98(0.64, 1.49) |  | 1.01(0.65, 1.58) |  |
|  | *Q3* | 0.91(0.60, 1.39) |  | 0.95(0.59, 1.53) |  | 1.05(0.65, 1.70) |  | 1.08(0.64, 1.81) |  |
|  | *Q4* | 0.62(0.38, 1.01) |  | 0.62(0.35, 1.07) |  | 0.69(0.40, 1.20) |  | 0.72(0.41, 1.26) |  |
|  | **OBS(median)** |  | <0.054 |  | <0.13 |  | <0.4 |  | <0.4 |
|  | *Pro-oxidative* | — |  | — |  | — |  | — |  |
|  | *Anti-oxidative* | 0.80(0.63, 1.01) |  | 0.81(0.60, 1.08) |  | 0.88(0.65, 1.19) |  | 0.89(0.65, 1.21) |  |
| **AF<14** | **OBS(quartile)** |  | **<0.001***** |  | **<0.001***** |  | **<0.001***** |  | **<0.001***** |
|  | *Q1* | — |  | — |  | — |  | — |  |
|  | *Q2* | 0.84(0.62, 1.13) |  | 0.85(0.59, 1.22) |  | 0.93(0.61, 1.42) |  | 0.95(0.61, 1.50) |  |
|  | *Q3* | 0.43(0.30, 0.62) |  | 0.47(0.33, 0.67) |  | 0.55(0.37, 0.81) |  | 0.56(0.36, 0.85) |  |
|  | *Q4* | 0.47(0.30, 0.73) |  | 0.53(0.34, 0.83) |  | 0.68(0.41, 1.11) |  | 0.68(0.40, 1.18) |  |
|  | **OBS(median)** |  | **<0.001***** |  | **<0.001***** |  | **<0.001***** |  | **<0.001***** |
|  | *Pro-oxidative* | — |  | — |  | — |  | — |  |
|  | *Anti-oxidative* | 0.50(0.39, 0.64) |  | 0.54(0.43, 0.68) |  | 0.63(0.50, 0.80) |  | 0.63(0.49, 0.81) |  |
| ^1^OR = Odds Ratio, CI = Confidence Interval | | | | | | | | | |
| *P < 0.05,**P<0.01,***P<0.001. | | | | | | | | | |
