# Supplementary material for: The interaction between sleep patterns and oxidative balance scores on the risk of cognitive function decline: Results from the national health and nutrition examination survey 2011–2014
Source: PLoS One. 2024 Dec 27;19(12):e0313784. doi: 10.1371/journal.pone.0313784 (PMC11676575; doi:10.1371/journal.pone.0313784)
Supplement: S7 Table — (DOCX) [file pone.0313784.s007.docx]

| **Table S7. Weighted odds ratios with 95% CI of logistic regression analyses of the association between combined sleep patterns and OBS and PCP.** | | | | | | | | | |
| --- | --- | --- | --- | --- | --- | --- | --- | --- | --- |
| **Characteristic** | | **Crude model** | | **Model 1** | | **Model 2** | | **Model 3** | |
|  |  | **OR**^1^(**95% CI**^1^) | ***P*-value** | **OR**^1^(**95% CI**^1^) | ***P*-value** | **OR**^1^(**95% CI**^1^) | ***P*-value** | **OR**^1^(**95% CI**^1^) | ***P*-value** |
| **DSST<34** | **Sleep duration+OBS** |  | **<0.001***** |  | **<0.001***** |  | **0.002*** |  | **0.002*** |
|  | *Normal Sleep duration+anti-oxidant OBS* | — |  | — |  | — |  | — |  |
|  | *Normal Sleep duration+pro-oxidant OBS* | 2.31(1.55, 3.44) |  | 2.16(1.48, 3.16) |  | 1.46(0.94, 2.27) |  | 1.48(0.93, 2.34) |  |
|  | *Short Sleep duration+anti-oxidant OBS* | 1.29(0.79, 2.09) |  | 1.25(0.72, 2.18) |  | 1.02(0.51, 2.03) |  | 1.00(0.46, 2.17) |  |
|  | *Short Sleep duration+pro-oxidant OBS* | 2.82(2.01, 3.95) |  | 2.15(1.46, 3.15) |  | 1.41(0.86, 2.32) |  | 1.22(0.70, 2.11) |  |
|  | *Long Sleep duration+anti-oxidant OBS* | 1.44(0.79, 2.63) |  | 1.26(0.65, 2.45) |  | 0.90(0.47, 1.73) |  | 0.91(0.42, 1.95) |  |
|  | *Long Sleep duration+pro-oxidant OBS* | 6.03(3.11, 11.7) |  | 5.66(3.01, 10.6) |  | 4.50(1.99, 10.2 |  | 4.34(1.71, 11.0) |  |
|  | **Sleep disorder+OBS** |  | **<0.001***** |  | **<0.001***** |  | **0.001***** |  | **0.002*** |
|  | *Normal Sleep +anti-oxidant OBS* | — |  | — |  | — |  | — |  |
|  | *Sleep Disorder +anti-oxidant OBS* | 1.20(0.57, 2.52) |  | 1.48(0.69, 3.17) |  | 1.75(0.76, 4.05) |  | 1.43(0.56, 3.62) |  |
|  | *Normal Sleep +pro-oxidant OBS* | 2.69(1.93, 3.76) |  | 2.42(1.80, 3.27) |  | 1.87(1.32, 2.65) |  | 1.81(1.25, 2.63) |  |
|  | *Sleep Disorder +pro-oxidant OBS* | 1.78(1.22, 2.60) |  | 1.99(1.36, 2.91) |  | 1.54(0.93, 2.54) |  | 1.25(0.71, 2.20) |  |
| **CERAD-WL<17** | **Sleep duration+OBS** |  | **<0.001***** |  | **<0.001***** |  | **0.013*** |  | **0.018*** |
|  | *Normal Sleep duration+anti-oxidant OBS* | — |  | — |  | — |  | — |  |
|  | *Normal Sleep duration+pro-oxidant OBS* | 1.65(1.25, 2.18) |  | 1.70(1.22, 2.38) |  | 1.36(1.00, 1.85) |  | 1.37(0.97, 1.93) |  |
|  | *Short Sleep duration+anti-oxidant OBS* | 1.07(0.66, 1.72) |  | 1.18(0.71, 1.98) |  | 1.06(0.59, 1.90) |  | 1.07(0.56, 2.05) |  |
|  | *Short Sleep duration+pro-oxidant OBS* | 1.87(1.30, 2.68) |  | 1.92(1.30, 2.83) |  | 1.46(1.00, 2.15) |  | 1.43(0.90, 2.25) |  |
|  | *Long Sleep duration+anti-oxidant OBS* | 1.24(0.70, 2.22) |  | 1.00(0.53, 1.87) |  | 0.86(0.44, 1.68) |  | 0.84(0.38, 1.89) |  |
|  | *Long Sleep duration+pro-oxidant OBS* | 3.24(1.84, 5.71) |  | 3.19(1.57, 6.47) |  | 2.66(1.26, 5.61) |  | 2.65(1.14, 6.16) |  |
|  | **Sleep disorder+OBS** |  | **<0.001***** |  | **<0.001***** |  | **<0.007**** |  | **<0.007**** |
|  | *Normal Sleep +anti-oxidant OBS* | — |  | — |  | — |  | — |  |
|  | *Sleep Disorder +anti-oxidant OBS* | 0.63(0.32, 1.28) |  | 0.67(0.32, 1.41) |  | 0.68(0.29, 1.60) |  | 0.65(0.25, 1.71) |  |
|  | *Normal Sleep +pro-oxidant OBS* | 1.73(1.30, 2.30) |  | 1.75(1.25, 2.45) |  | 1.46(1.05, 2.02) |  | 1.44(1.00, 2.06) |  |
|  | *Sleep Disorder +pro-oxidant OBS* | 1.67(1.06, 2.63) |  | 1.87(1.14, 3.08) |  | 1.54(0.87, 2.73) |  | 1.54(0.80, 2.94) |  |
| **CERAD-DR<5** | **Sleep duration+OBS** |  | **<0.001***** |  | **<0.001***** |  | **<0.001***** |  | **<0.001***** |
|  | *Normal Sleep duration+anti-oxidant OBS* | — |  | — |  | — |  | — |  |
|  | *Normal Sleep duration+pro-oxidant OBS* | 1.31(0.93, 1.84) |  | 1.29(0.87, 1.92) |  | 1.18(0.80, 1.75) |  | 1.18(0.75, 1.84) |  |
|  | *Short Sleep duration+anti-oxidant OBS* | 0.73(0.43, 1.24) |  | 0.78(0.44, 1.39) |  | 0.75(0.40, 1.39) |  | 0.76(0.38, 1.52) |  |
|  | *Short Sleep duration+pro-oxidant OBS* | 0.94(0.65, 1.35) |  | 0.92(0.58, 1.46) |  | 0.81(0.48, 1.35) |  | 0.78(0.44, 1.37) |  |
|  | *Long Sleep duration+anti-oxidant OBS* | 2.37(1.28, 4.39) |  | 2.02(0.99, 4.13) |  | 1.94(0.91, 4.14) |  | 1.9(0.80, 4.52) |  |
|  | *Long Sleep duration+pro-oxidant OBS* | 2.55(1.46, 4.46) |  | 2.35(1.22, 4.52) |  | 2.13(1.05, 4.33) |  | 2.08(0.95, 4.54) |  |
|  | **Sleep disorder+OBS** |  | 0.2 |  | 0.4 |  | 0.7 |  | 0.7 |
|  | *Normal Sleep +anti-oxidant OBS* | — |  | — |  | — |  | — |  |
|  | *Sleep Disorder +anti-oxidant OBS* | 0.75(0.40, 1.38) |  | 0.79(0.41, 1.54) |  | 0.82(0.41, 1.64) |  | 0.78(0.37, 1.65) |  |
|  | *Normal Sleep +pro-oxidant OBS* | 1.26(0.97, 1.63) |  | 1.25(0.90, 1.74) |  | 1.15(0.81, 1.63) |  | 1.13(0.78, 1.63) |  |
|  | *Sleep Disorder +pro-oxidant OBS* | 1.03(0.60, 1.79) |  | 1.03(0.59, 1.80) |  | 0.95(0.54, 1.69) |  | 0.93(0.51, 1.71) |  |
| **AF<14** | **Sleep duration+OBS** |  | **<0.001***** |  | **<0.001***** |  | **<0.001***** |  | **<0.001***** |
|  | *Normal Sleep duration+anti-oxidant OBS* | — |  | — |  | — |  | — |  |
|  | *Normal Sleep duration+pro-oxidant OBS* | 2.03(1.44, 2.86) |  | 1.95(1.30, 2.92) |  | 1.64(1.06, 2.56) |  | 1.68(1.02, 2.74) |  |
|  | *Short Sleep duration+anti-oxidant OBS* | 1.29(0.85, 1.95) |  | 1.27(0.76, 2.13) |  | 1.17(0.68, 2.03) |  | 1.18(0.63, 2.21) |  |
|  | *Short Sleep duration+pro-oxidant OBS* | 2.76(1.98, 3.84) |  | 2.19(1.55, 3.10) |  | 1.81(1.22, 2.67) |  | 1.74(1.13, 2.70) |  |
|  | *Long Sleep duration+anti-oxidant OBS* | 2.32(1.38, 3.91) |  | 2.13(1.27, 3.57) |  | 2.05(1.12, 3.77) |  | 2.04(1.03, 4.05) |  |
|  | *Long Sleep duration+pro-oxidant OBS* | 3.78(1.90, 7.55) |  | 3.41(1.74, 6.66) |  | 2.93(1.40, 6.11) |  | 2.99(1.34, 6.66) |  |
|  | **Sleep disorder+OBS** |  | **<0.001***** |  | **<0.001***** |  | **<0.001***** |  | **<0.001***** |
|  | *Normal Sleep +anti-oxidant OBS* | — |  | — |  | — |  | — |  |
|  | *Sleep Disorder +anti-oxidant OBS* | 0.80(0.34, 1.85) |  | 0.86(0.36, 2.04) |  | 0.89(0.36, 2.21) |  | 0.84(0.31, 2.26) |  |
|  | *Normal Sleep +pro-oxidant OBS* | 2.06(1.57, 2.70) |  | 1.85(1.45, 2.35) |  | 1.59(1.23, 2.04) |  | 1.58(1.20, 2.08) |  |
|  | *Sleep Disorder +pro-oxidant OBS* | 1.51(0.93, 2.44) |  | 1.63(0.93, 2.86) |  | 1.43(0.75, 2.74) |  | 1.41(0.72, 2.80) |  |
| ^1^OR = Odds Ratio, CI = Confidence Interval | | | | | | | | | |
| *P < 0.05,**P<0.01,***P<0.001. | | | | | | | | | |
