# Supplementary material for: The interaction between sleep patterns and oxidative balance scores on the risk of cognitive function decline: Results from the national health and nutrition examination survey 2011–2014
Source: PLoS One. 2024 Dec 27;19(12):e0313784. doi: 10.1371/journal.pone.0313784 (PMC11676575; doi:10.1371/journal.pone.0313784)
Supplement: S8 Table — (DOCX) [file pone.0313784.s008.docx]

| **Table S8. Weighted odds ratios with 95% CI of stratified analyses of the associations of anti-oxidative OBS with PCP compared with pro-oxidant OBS.** | | | | | | | | | |
| --- | --- | --- | --- | --- | --- | --- | --- | --- | --- |
| **Characteristic** | | **Crude model** | | **Model 1** | | **Model 2** | | **Model 3** | |
|  |  | **OR**^1^(**95% CI**^1^) | ***P*-value** | **OR**^1^(**95% CI**^1^) | ***P*-value** | **OR**^1^(**95% CI**^1^) | ***P*-value** | **OR**^1^(**95% CI**^1^) | ***P*-value** |
| **DSST<34** | **Sleep duration** |  |  |  |  |  |  |  |  |
|  | *Normal(7-8h)* | 0.43(0.29, 0.65) | **<0.001***** | 0.47(0.32, 0.70) | 0.76 | 0.69(0.45, 1.08) | 0.76 | 0.67(0.44, 1.03) | **0.04*** |
|  | *Short Sleep(≤6h)* | 0.46(0.29, 0.73) | **<0.001***** | 0.54(0.31, 0.96) | **0.027*** | 0.65(0.32, 1.32) | 0.2 | 0.72(0.33, 1.60) | 0.4 |
|  | *Long Sleep(≥9h)* | 0.24(0.11, 0.53) | **<0.001***** | 0.24(0.11, 0.55) | **<0.001***** | 0.21(0.06, 0.75) | **0.009**** | 0.22(0.06, 0.77) | **0.007**** |
|  | **Sleep disorder** |  |  |  |  |  |  |  |  |
|  | *Yes* | 0.67(0.35, 1.31) | 0.2 | 0.78(0.37, 1.61) | 0.5 | 1.17(0.50, 2.72) | 0.7 | 1.51(0.63, 3.58) | 0.3 |
|  | *No* | 0.37(0.27, 0.52) | **<0.001***** | 0.42(0.31, 0.56) | **<0.001***** | 0.54(0.38, 0.76) | **<0.001***** | 0.56(0.39, 0.80) | **<0.001***** |
| **CERAD-WL<17** | **Sleep duration** |  |  |  |  |  |  |  |  |
|  | *Normal(7-8h)* | 0.61(0.46, 0.80) | **<0.001***** | 0.59(0.42, 0.82) | **<0.001***** | 0.71(0.53, 0.96) | **0.016*** | 0.71(0.53, 0.95) | **0.009**** |
|  | *Short Sleep(≤6h)* | 0.57(0.32, 1.01) | **0.046*** | 0.64(0.34, 1.19) | 0.13 | 0.75(0.39, 1.44) | 0.3 | 0.84(0.41, 1.69) | 0.6 |
|  | *Long Sleep(≥9h)* | 0.38(0.20, 0.74) | **0.003**** | 0.32(0.15, 0.66) | **<0.001***** | 0.31(0.13, 0.75) | **0.005**** | 0.28(0.10, 0.77) | **0.005**** |
|  | **Sleep disorder** |  |  |  |  |  |  |  |  |
|  | *Yes* | 0.38(0.19, 0.75) | **0.004**** | 0.35(0.14, 0.86) | **0.015*** | 0.44(0.15, 1.26) | 0.094 | 0.45(0.14, 1.44) | 0.13 |
|  | *No* | 0.58(0.43, 0.77) | **<0.001***** | 0.57(0.41, 0.79) | **<0.001***** | 0.69(0.50, 0.95) | **0.012*** | 0.70(0.50, 1.00) | **0.022*** |
| **CERAD-DR<5** | **Sleep duration** |  |  |  |  |  |  |  |  |
|  | *Normal(7-8h)* | 0.77(0.55, 1.07) | 0.11 | 0.78(0.52, 1.16) | 0.2 | 0.82(0.55, 1.23) | 0.3 | 0.84(0.55, 1.27) | 0.3 |
|  | *Short Sleep(≤6h)* | 0.78(0.45, 1.36) | 0.4 | 0.93(0.52, 1.68) | 0.8 | 1.05(0.54, 2.02) | 0.9 | 1.22(0.61, 2.44) | 0.5 |
|  | *Long Sleep(≥9h)* | 0.93(0.41, 2.12) | 0.9 | 0.79(0.33, 1.90) | 0.6 | 0.84(0.35, 2.02) | 0.7 | 0.74(0.31, 1.74) | 0.4 |
|  | **Sleep disorder** |  |  |  |  |  |  |  |  |
|  | *Yes* | 0.72(0.35, 1.51) | 0.4 | 0.76(0.33, 1.75) | 0.5 | 0.95(0.38, 2.37) | >0.9 | 0.92(0.31, 2.69) | 0.9 |
|  | *No* | 0.80(0.61, 1.03) | 0.073 | 0.80(0.58, 1.10) | 0.2 | 0.86(0.61, 1.21) | 0.3 | 0.88(0.62, 1.25) | 0.4 |
| **AF<14** | **Sleep duration** |  |  |  |  |  |  |  |  |
|  | *Normal(7-8h)* | 0.49(0.35, 0.69) | **<0.001***** | 0.51(0.34, 0.78) | **0.001**** | 0.59(0.37, 0.92) | **0.011*** | 0.59(0.37, 0.93) | **0.010*** |
|  | *Short Sleep(≤6h)* | 0.47(0.33, 0.67) | **<0.001***** | 0.54(0.39, 0.76) | **<0.001***** | 0.61(0.43, 0.87) | **0.003**** | 0.66(0.45, 0.99) | **0.019*** |
|  | *Long Sleep(≥9h)* | 0.61(0.24, 1.54) | 0.3 | 0.61(0.23, 1.58) | 0.3 | 0.71(0.27, 1.83) | 0.4 | 0.68(0.27, 1.73) | 0.4 |
|  | **Sleep disorder** |  |  |  |  |  |  |  |  |
|  | *Yes* | 0.53(0.23, 1.24) | 0.13 | 0.55(0.21, 1.45) | 0.2 | 0.67(0.25, 1.76) | 0.4 | 0.68(0.24, 1.87) | 0.4 |
|  | *No* | 0.49(0.37, 0.64) | **<0.001***** | 0.54(0.42, 0.69) | **<0.001***** | 0.63(0.50, 0.81) | **<0.001***** | 0.64(0.49, 0.84) | **<0.001***** |
| ^1^OR = Odds Ratio, CI = Confidence Interval | | | | | | | | | |
| *P < 0.05,**P<0.01,***P<0.001. | | | | | | | | | |
