# Supplementary material for: The interaction between sleep patterns and oxidative balance scores on the risk of cognitive function decline: Results from the national health and nutrition examination survey 2011–2014
Source: PLoS One. 2024 Dec 27;19(12):e0313784. doi: 10.1371/journal.pone.0313784 (PMC11676575; doi:10.1371/journal.pone.0313784)
Supplement: Attachment — Submitted filename: Response to Reviewers.docx [file pone.0313784.s009.docx]

# Response to Reviewers

Dear Esteemed Reviewers and Editor,

I hope this message finds you well.

I am writing to express my heartfelt gratitude for the valuable feedback from you, the esteemed reviewers and editor, regarding my manuscript titled “The Interaction Between Sleep Patterns and Oxidative Balance Scores on the Risk of Cognitive Function Decline: Results from the National Health and Nutrition Examination Survey2011-2014.” I am truly thankful for your dedication and insightful comments.

In response to your suggestions, I have made the following revisions to the manuscript:

1. In accordance with PLOS ONE’s formatting requirements, I have revised the overall format of the manuscript, including author attribution, title formatting and size, citation format for figures and tables, and reference formatting. Additionally, we made adjustments to the table format to ensure that the headers repeat across pages for the reader's convenience. If you notice any remaining formatting inconsistencies in the revised manuscript, please do not hesitate to contact me, and I will be more than happy to correct any errors.
2. I have moved the ethics statement to the methodology section.
3. We have removed the table from the manuscript that was also repeated in the supplementary materials.
4. I have checked and updated the references to ensure that there are no retracted publications included.
5. In the introduction, I enhanced the discussion of the oxidative balance score (OBS) and clearly presented the hypotheses underpinning our research questions, thereby improving the logical flow of the paper.
6. I have removed the abbreviations from the keywords, except for “NHANES,” as we noted that many other articles have adopted a similar approach.
7. In the discussion section, I have provided additional information pertaining to the limitation regarding “self-reported data may be subjective” and offered potential methods for addressing this limitation.

Finally, these are the numerous revisions made to the manuscript. I sincerely appreciate the diligence of all reviewers and kindly request any further feedback you may have on our manuscript.

Warm regards,

Taiwei Lou
